# Evaluation of the uptake, retention and effectiveness of exercise referral schemes for the management of mental health conditions in primary care: a systematic review

**DOI:** 10.1186/s12889-022-12638-7

**Published:** 2022-02-07

**Authors:** Samuel Tomlinson-Perez, Katarzyna Karolina Machaczek, Joseph Firth, Nicholas Pollard, Goutham Meda, Ellis Keddie, Elizabeth Goyder

**Affiliations:** 1 School of Medicine, University of Liverpool, Liverpool, UK; 2 College of Health, Wellbeing and Life Sciences, Sheffield Hallam University, Sheffield, UK; 3 Division of Psychology and Mental Health, University of Manchester, Manchester, UK; 4 Department of Allied Health Professionals, Sheffield Hallam University, Sheffield, UK; 5 School of Health and Related Research (ScHARR), University of Sheffield, Sheffield, UK

**Keywords:** Mental health, Anxiety, Depression, Physical activity, Exercise referral schemes, Uptake, Adherence, Effectiveness

## Abstract

**Background:**

Exercise is a recognised element of health-care management of mental-health conditions. In primary health care, it has been delivered through exercise referral schemes (ERS). The National Institute for Health and Care Excellence has highlighted uncertainty regarding the effectiveness of ERS in improving exercise participation and health outcomes among those referred for mental-health reasons. This review aims, therefore, to evaluate ERSs for individuals who are referred specifically for mental-health reasons.

**Methods:**

Studies were reviewed that assessed the effectiveness of ERSs in improving initiation of and/or adherence to exercise and/or their effectiveness in improving long-term participation in exercise and health outcomes among primary care patients who had been referred to the scheme for mental-health reasons. The data were extracted and their quality assessed. Data were analysed through a narrative synthesis approach.

**Results:**

Nine studies met the eligibility criteria. Three assessed clinical effectiveness of the schemes, eight assessed ERS uptake and/or adherence to the exercise schedule, and two assessed the impact of the ERSs on long-term exercise levels. In one study, it was found that ERSs that were based in leisure centres significantly improved long-term symptoms in those who had been referred due to their mental ill health (P<0.05). ERSs that involved face-to-face consultations and telephone calls had the highest rates of mean uptake (91.5%) and adherence (71.7%), but a difference was observed between uptake/adherence in trials (86.8%/55.3%) and in routine practice (57.9%/37.2%). ERSs that included face-to-face consultations and telephone calls increased the amount of long-term physical activity that was undertaken by people who had been referred for mental-health reasons (P=0.003).

**Conclusions:**

Uptake and effectiveness of ERSs for mental health conditions was related to programme content and setting with more effective programmes providing both face-to-face and telephone consultations. Good uptake of yoga among those referred for mental health reasons suggests that mindful exercise options should be investigated further. Existing ERSs could be improved through application of individual tailoring and the provision of more face-to-face consultations, and social support. Further research is required to identify the types of ERSs that are most clinically effective for those with mental ill health.

**Supplementary Information:**

The online version contains supplementary material available at 10.1186/s12889-022-12638-7.

## Background

Up to 15% of the UK population may experience a mental-health disorder at any one time [1]. Depression and anxiety are two of the commonest conditions. Depression is a leading cause of disability globally and is the leading cause worldwide of disability and premature deaths in adults aged 18-44 [2]. It has a prevalence of 4.5% among UK adults and is characterised by constant low mood and/or the loss of enjoyment in the majority of their activities (i.e. anhedonia), and a range of related emotional, cognitive, physical and behavioural symptoms [2]. Approximately 25% of adults experience anxiety at some point in their lives [3]. Generalised anxiety disorder is characterised by disproportionate, pervasive, uncontrollable and widespread levels of worry, with potential somatic, cognitive and behavioural symptoms [3]. Anxiety and depression form one of the most common comorbidities [4]. Approximately 67% of those with depression are thought to have a comorbid anxiety. Similarly, 63% of those with a primary anxiety disorder are likely to have concurrent depression [4]. Findings from a recent UK survey suggest that the incidence of stress has increased, with 74% of adults reporting that they feel overwhelmed or unable to cope due to mental or emotional pressures [5]. Individuals may also have multiple mental health conditions at any given time.

Primary care plays a central role in the management of mental ill-health; up to 90% of depression and anxiety cases are managed in this setting [1]. Numerous management methods are available in primary care for mental health conditions, including lifestyle advice, medication and psychotherapy. Increasing physical activity levels is a common lifestyle recommendation for many health conditions, as it has been demonstrated to improve overall health outcomes, quality of life, functional capacity and mood [6]. Physical activity is defined as any skeletal body movement that requires energy expenditure. Exercise is a subset of physical activity that is planned, structured and repetitive, with the goal of maintaining or improving fitness levels [7]. More specifically for mental health, physical activity has been shown to be effective for stress [8], clinical depression and anxiety [9]. Meanwhile, individuals not participating in regular physical activity are twice as likely to display depression and anxiety symptoms [10]. Furthermore, physical inactivity contributes towards the high levels of cardiometabolic diseases observed in people with mental illness [11].

Despite the well-known health benefits of exercise [12], many people lead sedentary lives, this is particularly the case for those with mental ill health [13]. In England, for example, 34% of men and 42% of women do not achieve the recommended amount of weekly aerobic exercise (150 min of moderate activity or 75 min of vigorous activity) [14]. Additionally, 27% of adults exercise for less than 30 min a week and are thereby classified as inactive [15].

One way to increase activity levels among sedentary individuals is through exercise referral schemes (ERS). These consist of an assessment by primary care or allied health professionals, followed by referral to a physical activity specialist and/or service. The patient is advised on the type of physical activity that suits the specific needs of the individual and he or she is given the opportunity to take part in an exercise programme [16], often based in a leisure centre [17]. ERSs can be funded by commissioners for the rehabilitation and management of certain health conditions including myocardial infarctions, stroke, chronic heart failure, chronic obstructive pulmonary disease, lower back pain and depression [16]. Individuals with stress and anxiety are also eligible for the scheme [18]. There are currently no standardised protocols for how ERS programmes are delivered or the type of exercises that are involved. This means a variety of ERSs are offered in the UK and there are currently no set guidelines for the type of ERSs that should be used in patients referred for mental health reasons. A lack of evidence regarding effectiveness for specific schemes or population subgroups is the primary cause for this [16]. It is, however, important to stress that a lack of standardisation in ERSs for mental health referrals is not necessarily negative, with increased individualisation of exercise programmes shown to improve engagement in this clinical population [19].

A preliminary literature search highlighted a gap in evidence regarding the effectiveness of ERSs on mental health, and this is supported by the latest National Institute for Health and Care Excellence (NICE) guidance on ERSs [16]. Previous reviews have assessed mental health outcomes in ERSs as part of a wider review scope [20, 21], but none have focused on mental health specifically. Additionally, many of the studies included participants referred for non-mental health reasons in the assessment of mental health outcomes. To reliably evaluate ERSs as a management method for patients with mental illness, it is important to analyse the body of research which focuses on participants with mental health diagnoses as their primary reason for referral. The emphasis was placed on studies focusing on depression and anxiety since they are the most prevalent mental health disorders in the UK population [2, 3]. Other mental health conditions, such as stress or post-traumatic stress disorder were also included in the review.

This review also explored the suitability of ERSs as an intervention in the real world by examining uptake and adherence. Both at the individual and population level, uptake and adherence are an important aspect of ability to benefit from an intervention. Assessing whether ERSs influence long-term physical activity levels is another important measure of effectiveness in those referred for mental health reasons. No reviews were found that explored all of these areas.

The aim of this review was to evaluate the use of ERSs as a management method for individuals referred for mental health reasons in a primary care setting. To address this aim the following primary objectives were set:

1. To assess the clinical effectiveness of ERSs on mental health symptoms in participants referred for mental health reasons.
2. To assess levels of uptake and adherence to ERSs among participants referred for mental health reasons.
3. To assess the effects of ERSs on long-term physical activity levels in participants referred for mental health reasons.

The secondary aim of this review was to assess uptake of and adherence to exercise programmes in mental health referrals compared to non-mental health referrals in included studies.

## Methods

### Search strategy and study selection

Adhering to PRISMA guidelines [22], a literature search was conducted in five electronic databases: MEDLINE, PsycInfo, CINAHL, Scopus, and the Cochrane Library. A pre-specified review protocol was created for ERSs in mental health conditions (Additional File 1). The review was restricted to publications written in the English language, due to a lack of translation resources. Databases were searched from inception to July 2020. The search terms included ‘exercise’, ‘physical activity’, ‘referral’, ‘mental health’, ‘depression’, ‘mood disorders’, ‘affective disorders’, ‘anxiety’, and ‘anxiety disorders’. Detailed search strategies for all databases are presented in Table S1 (please see Additional file 2). Reference lists of relevant studies were scanned, and citation searches using Google Scholar were also undertaken. Deduplication was performed for all records identified. Titles and abstracts of remaining records were screened to exclude irrelevant studies. All remaining articles were read in full and selected for inclusion if they met the eligibility criteria. All titles and abstracts were reviewed by two independent reviewers (STP & GM) to determine appropriateness to the purpose of the review. Any disagreements over study inclusion were resolved by discussion. Similarly, the two reviewers reviewed full texts independently and compared these against predefined eligibility criteria to confirm the article’s appropriateness for inclusion in the review.

### Eligibility Criteria

The population, intervention, comparator, outcome (PICO) framework [23] was used to clarify inclusion criteria (please see Table 1). All quantitative study designs were eligible. Studies were excluded if mental health was not specified as a primary referral reason, they included individuals participating in regular exercise, or evaluated exercise interventions that did not meet ERS criteria. No limits were placed on duration or severity of conditions, or on age and medication use. No restrictions were placed on the type of tools used to measure outcomes or outcome assessment timings.

**Table 1:** Inclusion criteria based on PICO framework

***Inclusion Criteria***	
**1.** Study participants were diagnosed with a mental health condition, with primary care being the main source of referral.	
**2.** Mental health was the primary reason for referral.	
**3.** Studies evaluating ERS, as defined by Pavey et al. [21]	
o Referral by a primary-care health-care professional to a service designed to increase physical activity or exercise	
o Physical activity/exercise programme tailored to individual needs	
o Initial assessment and monitoring throughout the programme	
**4.** Studies with any relevant comparator were permitted.	
**5. **Studies had to measure one or more of the following	
o Changes in clinical symptoms of mental health conditions (e.g. depression and anxiety) found or managed in primary care	
o ERS uptake/adherence rates of individuals referred from primary care for mental health reasons	
o Impact of ERS on long-term physical activity levels in participants referred from primary care for mental health reasons.	

### Data extraction

The data extraction process was undertaken by two reviewers (STP & EK) using a piloted data extraction form. Recorded information included details of the studies (e.g. author, year, setting, study type), participants (e.g. sample size, age, gender, mental health conditions), details of intervention/comparators (e.g. type, length, frequency/duration of sessions), outcomes (e.g. primary/secondary outcomes, outcome measures, assessment timings), and results. Authors were contacted directly if there was insufficient data to evaluate the research findings in the published paper.

### Quality assessment

The risk of bias in all included articles was assessed at the study level, based on study design-specific criteria and conduct. The Cochrane Collaboration’s risk of bias tool [24] was used to analyse risk of bias in randomised controlled trials (RCTs). A risk of bias graph and summary were created for RCTs with Review Manager 5.3 software [25]. The other studies were treated as case series, and were assessed using a modified version of the Institute of Health Economics Quality Appraisal Checklist [26]. A quality appraisal checklist table was created. Two reviewers (STP & GM) independently performed quality assessment. Disagreements were resolved by discussion.

### Data synthesis

Due to considerable inter-study heterogeneity, performing a meta-analysis was deemed to be inappropriate. A narrative synthesis approach was used to analyse the results for each outcome. Results data were combined for both uptake and adherence outcomes (for mental health referrals, and mental health compared to non-mental health referrals). This was performed by calculating mean values across studies based on individual participant data. Mean values were calculated for different ERS types and different study settings.

## Results

### Study selection

**Fig. 1:**
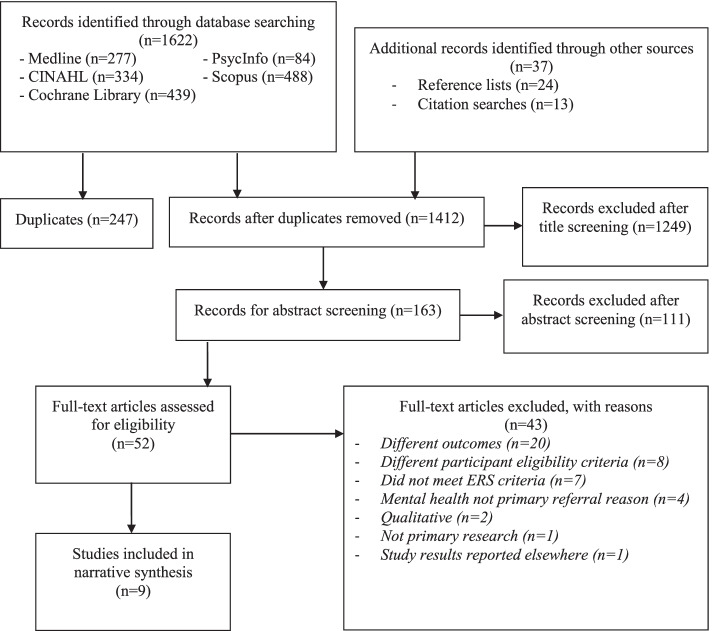
Modified PRISMA flowchart of literature search results

A total of 1659 records were retrieved from database searches, of which 257 records were duplicates. A further 1360 were excluded following screening of titles and abstracts (Fig. 1). After full-text screening of 52 articles, 9 manuscripts [27–35] were included in this review (Additional file 3).

### Study characteristics

Characteristics of the nine included [27–35] studies are shown in Table 2. Data from Murphy et al. [30] and Moore et al. [31] originated from the same trial but since they presented different outcome measures, they are referred to as separate studies.

**Table 2:** Study characteristics

First Author Year Country	Study Type	MH Eligibility Criteria	Mental health condition (% of patients)	Includes Non-MH Referrals (Yes/No)	Comparator(s)	Sample size (ERS/C if applicable) Female % (ERS/C if applicable)	Mean age (± SD) ERS/C if applicable	Outcomes of this review addressed
Harrison [27] 2005 United Kingdom	Retrospective data analysis	Participant characteristics: - ≥18 years old - Sedentary Referral reasons: - MH (types not specified)	N/I	Yes	Participants referred for non-MH reasons (took part in same ERS programme)	Sample size = 339 Total female (%) = 60.8	Total = 51.3 (±12.6)	- Uptake/ Adherence
Crone [28] 2008 United Kingdom	Retrospective data analysis	Participant characteristics: - N/I Referral reasons: - Depression - Anxiety/ Loss of confidence - Stress/ Tension	Anxiety/loss of confidence = 26% Depression = 61% Stress/tension = 13%	Yes	Participants referred for physical health conditions (took part in same ERS programme)	Sample size = 134 Female (%) = 64	42 (±14)	- Uptake/ Adherence
Chalder [29] 2012 United Kingdom	RCT	Participant characteristics: - 18-69 years old - Have not failed to respond to antidepressants Referral reasons: - Mild/moderate depression	Depression = 100%	No	Control group who received usual care	Sample size = 361 (182/179) Female (%) = 68/65	40.9 (±12.5) / 38.8 (±12.7)	- Clinical effectiveness - Uptake/ Adherence - Long-term activity levels
Murphy [30] 2012 United Kingdom	RCT	Participant characteristics: - >16 years old - Sedentary Referral reasons: - Mild anxiety - Mild depression - Stress	Anxiety = N/I Depression = N/I Stress = N/I	Yes	Control group who received usual care, information leaflet and addresses of local facilities	Sample size = 601 (310/291) - MH only = 79 (41/38) - MH+CHD = 522 (269/253) Female (%) = 65.6/65.5	Total = 52 (±14.7)	- Clinical effectiveness - Long-term activity levels
Moore [31] 2013 United Kingdom	RCT	Participant characteristics: - >16 years old - Sedentary Referral reasons: - Mild anxiety - Mild depression - Stress	Anxiety = N/I Depression = N/I Stress = N/I	Yes	Participants referred for coronary heart disease only (took part in same ERS programme)	Sample size = 310 MH: - MH only = 41 - MH+CHD = 269 Total female (%) = 65.6	Total = 52 (±14.7)	- Uptake/ Adherence
Forsyth [32] 2015 Australia	RCT	Participant characteristics: - >18 years old Referral reasons: - Depression - Anxiety	Depression only = 46% Anxiety only = 25% Both = 29%	No	Control group received 5-minute phone call every two weeks to assess for any diet or physical activity changes. No advice given	Sample size = 94 (52/42) Female (%) = 73/71	43.88 (±14.17)^a^ / 47.33 (±13.45)^a^	- Clinical effectiveness - Uptake/ Adherence
Tobi [33] 2017 United Kingdom	Retrospective data analysis	Participant characteristics: - Sedentary Referral reasons: - Depression - Anxiety - Stress - Other mental disorders	Anxiety = N/I Depression = N/I Stress = N/I Other mental disorders = N/I	Yes	Participants referred for physical health conditions (took part in same ERS programme)	Sample size = 141 Female (%) = 63.1	41.3 (±11.8)	- Uptake/ Adherence
Avery [34] 2020 United States of America	Retrospective data analysis	Participant characteristics: - War veterans Referral reasons: - Depression - Stress/anxiety - Post-traumatic stress disorder	Depression = 3%^a^ Stress/anxiety = 95%^a^ Post-traumatic stress disorder = 3%^a^	Yes	Participants referred for physical health conditions (took part in same ERS programme)	Sample size = 149^a^ Female (%) = 70.1%	58.4 (±14.4)^a^	- Uptake/ Adherence
Morgan [35] 2020 United Kingdom	Retrospective data linkage study	Participant characteristics: - >16 years old - Sedentary Referral reasons: - Mild anxiety - Mild depression - Stress	Anxiety = N/I Depression = N/I Stress = N/I	Yes	Participants referred for coronary heart disease only (took part in same ERS programme)	Sample size = 14,632 - MH only = 8603 - MH+CHD = 6029 Total female (%) = 61.65	Total = 53 (±16.6)	- Uptake/ Adherence

^a^ = information obtained from author, *N/I *no information provided, *MH* mental health, *ERS* exercise referral scheme, *C* comparator, *SD* standard deviation, *RCT* randomised controlled trial, *CHD* coronary heart disease

The most common study type was a retrospective analysis of ERS data [27, 28, 33–35]; or an RCT [29–32]. Studies targeted patients treated for mental ill health [29, 32] or mental ill health and other chronic health conditions [27, 28, 30, 31, 35].

Six studies assessed leisure centre-based ERSs [27, 28, 30, 31, 33, 35]. Three of these [30, 31, 35] provided access to leisure facilities and exercise sessions throughout, two in-person consultations, plus one telephone call. One [27] provided the same but without telephone contact. One [28] required participants to attend biweekly exercise classes. One [33] provided educational classes and access to exercise sessions, although no information was provided on frequency or duration.

Two studies assessed ERSs involving regular 30- to 60-minute face-to-face consultations and telephone calls with exercise professionals [29, 32]. These meetings aimed to motivate and educate participants to increase physical activity levels. One study assessed an ERS involving lifelong referral to 60-minute therapeutic yoga classes with up to eight sessions a week [34]. Apart from lifelong yoga referrals [34], all ERSs lasted 8-32 weeks. The characteristics of interventions are listed in Table 3.

**Table 3:** Study intervention details

Study	Length	Type of ERS	Frequency of sessions	Duration of sessions
Harrison 2005 [27]	12 weeks	Leisure centre-based: - Consultations with exercise officer - Access to leisure facilities and supervised exercise sessions	- Access to leisure facilities and exercise sessions throughout (encouraged to attend ≥2 sessions/week - Two consultations (weeks 0, 12)	N/I
Crone 2008 [28]	8-12 weeks	Leisure centre-based: - Gym sessions (majority) - Swimming - Circuits - Exercise-to-music classes	Twice a week	N/I
Chalder 2012 [29]	32 weeks	Regular face-to-face consultations and telephone calls with physical activity facilitator	Participants organise timing of: - Three face to face consultations - Ten telephone contacts	30-60 min
Murphy 2012 [30]	16 weeks	Leisure centre-based: - Face to face consultations & telephone contact with exercise professional - Access to one to one exercise instruction & group classes	- Access to exercise instruction and classes throughout - Two consultations (weeks 0, 16) - One telephone contact (week 4)	N/I
Moore 2013 [31]	16 weeks	Leisure centre-based: - Face to face consultations & telephone contact with exercise professional - Access to one to one exercise instruction & group classes	- Access to exercise instruction and classes throughout - Two consultations (weeks 0, 16) - One telephone contact (week 4)	N/I
Forsyth 2015 [32]	12 weeks	Regular face-to-face consultations and telephone calls with dietician/exercise physiologists	Once every two weeks	30-60 min^a^
Tobi 2017 [33]	20-26 weeks	Leisure centre-based: - Motivational/educational classes - Access to group exercise - Healthy walks - Gym sessions - Swimming/water workouts	N/I	N/I
Avery 2020 [34]	Lifelong referral	Therapeutic yoga classes in person and via video link with yoga instructor	Up to 8 classes a week	60 min
Morgan 2020 [35]	16 weeks	Leisure centre-based: - Face to face consultations & telephone contact with exercise professional - Access to one to one exercise instruction & group classes	- Access to exercise instruction and classes throughout - Two consultations (weeks 0, 16) - One telephone contact (week 4)	N/I

^a^ = information obtained from author, *N/I* No information provided, *ERS* exercise referral scheme

### Quality assessment

Additional file 4 provides further details about quality assessment. Figures S1 and S2 show the risk of bias graph and summary created for the four RCTs [29–32]. Risk of selection bias was low for two [30, 31] and unclear for two studies [29, 32]. Random sequence generation was achieved using a random number generator [30, 31] or randomisation chart [32]. Allocation concealment was ensured by allocating treatment remotely [30, 31], or using an automated telephone system [29]. All included trials were at high risk of performance bias because the nature of the intervention made it impossible to blind participants. Three studies were deemed to be at low risk of detection bias [30–32]. All studies scored at high risk of attrition bias. The evidence for reporting bias was not found.

Table S2 (please see Additional File 4) shows the quality appraisal checklist for the five case series studies [27, 28, 33–35]. A potential risk of bias for all studies was due to their retrospective nature. Other sources of bias included not knowing whether patients were recruited consecutively, and whether severity of mental health conditions in participants was similar.

### Primary outcomes

Primary outcomes of this review were to assess clinical effectiveness of ERSs on mental health symptoms, uptake and adherence of ERS participants referred for mental health reasons, and effects of ERSs on long-term physical activity levels in mental health patients. Tables have been created for all primary outcomes (Tables 4, 5 and 6). Each table displays individual results and participant characteristics for every study pertinent to the respective outcome.

**Table 4:** Clinical effectiveness of ERS on mental health symptoms

Study details	Outcome measure	Outcome assessment timings	Short-term results ERS vs. C	Long-term results ERS vs. C
Chalder 2012 [29] Trial	BDI-II	4, 8, 12 months	4 months adjusted between group difference in mean BDI-II score = -0.54 (95%CI -3.06 to 1.99) P=0.68	Combined 4, 8 and 12 months adjusted between group difference in mean BDI-II score = -1.20 (95%CI -3.42 to 1.02) P=0.29
Murphy 2012 [30] Trial	HADS	12 months	Did not assess	HADS depression 12 months adjusted between group difference in HADS depression score = -1.39 (95%CI -2.60 to -0.18) P<0.05 HADS anxiety 12 months adjusted between group difference in HADS anxiety score = -1.56 (95%CI -2.75 to -0.38) P<0.05
Forsyth 2015 [32] Trial	DASS-21	3 months	DASS-21 depression subscale ERS difference from baseline = -2.1 C difference from baseline = -4.0 Between group difference P=0.1 DASS-21 anxiety subscale ERS difference from baseline = -1.4 C difference from baseline = -3.0 Between group difference P=0.08 DASS-21 stress subscale ERS difference from baseline = -1.5 C difference from baseline = -1.8 Between group difference P=0.06 Total DASS-21 scores ERS difference from baseline = -5.1 C difference from baseline = -6.1 Between group difference P=0.04	Did not assess

*ERS* exercise referral scheme group, *C* comparator group, *CI* confidence interval, *HADS* Hospital Anxiety and Depression Scale, *BDI-II* Beck depression inventory (version II) score, *DASS-21* Depression, Anxiety and Stress Scale, *P*<0.05 = significant difference

**Table 5:** ERS uptake and adherence in mental health referrals

Study details	ERS information	Outcome measure	Results 1. Uptake 2. Adherence	Results in non-MH referrals (if applicable) 1. Uptake 2. Adherence
Harrison 2005 [27] Routine practice	12 weeks leisure centre-based	Attended first appointment	1. Attended first appointment - MH = 280 (uptake 82.6%) 2. N/I	1. Attended first appointment - C = 4945 (uptake 78.9%) 2. N/I
Crone 2008 [28] Routine practice	8-12 weeks leisure centre-based	Uptake - Attended first session Completed - Attended ≥80% of scheduled sessions	1. Uptake - MH = 79 (uptake 59.0%) 2. Completed - MH = 29 (adherence 36.7%)	1. Uptake - C = 1917 (uptake 69.3%) 2. Completed - C = 935 (adherence 48.8%)
Chalder 2012 [29] Trial	32 weeks regular face-to-face consultations and telephone calls	Failed to attend - Did not attend first ERS session Received adequate dose - Had ≥5 sessions	1. Failed to attend - ERS group = 11 (uptake 94.5%) 2. Received adequate dose at 4 months - ERS group = 102 (59.6% adherence) Received adequate dose at 8 months - ERS group = 129 (75.4% adherence)	Did not assess
Moore 2013 [31] Trial	16 weeks leisure centre-based	Did not enter Partial attendance (0-16 weeks) Completed	1. Did not enter - MH = 52 (uptake 83.2%) 2. Partial attendance - MH = 152 Completed - MH = 106 (adherence 41.1%)	1. Did not enter - C = 109 (uptake 85.8%) Adjusted OR 0.82 (95%CI 0.57 to 1.17) 2. Partial attendance - C = 294 Completed - C = 367 (adherence 55.5%) Adjusted OR 0.57 (95%CI 0.43 to 0.75)
Forsyth 2015 [32] Trial	12 weeks regular face-to-face consultations and telephone calls	Declined referral Discontinued participation	1. Declined referral - ERS group = 9 (uptake 85.2%) 2. Discontinued participation - ERS group = 21 (adherence 59.6%)	Did not assess
Tobi 2017 [33] Routine practice	20-26 weeks leisure centre-based	Adherence - Attended ≥80% of scheduled sessions - Two recorded progress assessments	1. N/I 2. Adherence - MH = 53 (adherence 37.6%)	1. N/I 2. Adherence - C = 263 (adherence 47.0%) - Between group difference P = 0.04
Avery 2020 [34] Routine practice	Unlimited number of in person or video link yoga classes	Follow through/uptake - Attendance at ≥1 yoga class	1. Uptake - MH = 63 (uptake 42.3%) *Stress/anxiety (42%)* *Depression (40%)* *Post-traumatic stress disorder (45%)* 2. N/I	1. Uptake - C = 74 (uptake 27.1%) 2. N/I
Morgan 2020 [35] Routine practice	16 weeks leisure centre-based	Did not take up Uptake	1. Uptake - MH only = 4677 (54.4% uptake) - MH+CHD = 3730 (61.9% uptake) - All MH = 8407 (57.5% uptake) 2. N/I	1. Uptake - C = 10,699 (67.7% uptake) - OR 0.79, 95%CI 0.74 to 0.84 2. N/I

* = information obtained from author, *MH* mental health, *C* comparator, *ERS* exercise referral scheme, *N/I* no information available, *CHD* coronary heart disease, *SD* standard deviation, *OR* odds ratio, *CI* confidence interval, *P*<0.05 = significant difference

**Table 6:** Effects of ERS on long-term physical activity in mental health referrals

Study details	Outcome measure	Outcome assessment	Results ERS vs. C
Chalder 2012 [29] Trial	MET minutes of physical activity a week - Meeting current exercise guidelines if MET ≥1000	4, 8, 12 months	Participants doing ≥1000 MET minutes of physical activity per week (%): 4 months - ERS = 52% - C = 43% 8 months - ERS = 63% - C = 49% 12 months - ERS = 58% - C = 40% Between group difference at 4 months Adjusted OR 1.58 (95%CI 0.94 to 2.66) P = 0.08 Between group difference using combined 4-, 8- and 12-month data Adjusted OR 2.27 (95%CI 1.32 to 3.89) P = 0.003
Murphy 2012 [30] Trial	7-D PAR	12 months	12 months adjusted between group difference in 7D-PAR score = OR 1.06 (95%CI 0.73 to 1.55) P>0.05

*ERS* exercise referral scheme group, *C* comparator group, *SD* standard deviation, *MH* mental health, *CHD* coronary heart disease, *N/I* no information available, *CI* confidence interval, *7-D PAR* 7-day physical activity recall, *MET* metabolic equivalent of the task, *OR* odds ratio, *P*<0.05 = significant difference

### Clinical effectiveness on mental health symptoms

Three RCTs [29, 30, 32] assessed the clinical effectiveness of ERSs for mental health disorders (Table 4). Studies used a range of outcomes: Hospital Anxiety and Depression Scale (HADS); Beck Depression Inventory Version II Score (BDI-II); and Depression, Anxiety and Stress Scale (DASS-21).

When combining results from 4, 8 and 12 months, Chalder et al. [29] recorded a non-significant between group mean difference in the BDI-II score in favour of the intervention group. After adjusting for all covariates, Murphy et al. [30] found, at 12 months, that the ERS group had a significantly lower HADS anxiety (-1.56) and depression (-1.39) scores compared to the control group.

### Uptake and adherence in mental health referrals

Eight studies [27–29, 31–35] assessed this outcome (Table 5). Three of these were RCTs [29, 31, 32] and five were in routine practice [27, 28, 33–35].

#### Uptake

Seven studies [27–29, 31, 32, 34, 35] assessed uptake of ERSs amongst patients referred for mental health reasons. Four studies [27, 28, 31, 35] used a leisure centre-based ERS, with uptake ranging from 57.5% [35] to 83.2% [31]. Mean uptake in these four studies was 58.5%. Two studies [29, 32] used ERSs involving regular face-to-face consultations and telephone calls. Uptake levels in these studies were 94.5% [29] and 85.2% [32]. Mean uptake across both studies was 91.5%. One study [34] involved yoga classes and had an uptake of 42.3%.

#### Uptake in routine practice versus uptake in trials

Uptake of the scheme in routine practice ranged from 42.3 to 82.6% [27, 28, 34, 35]. The mean uptake across all four studies was 57.9%. The other study involved yoga classes [34] and had an uptake of 42.3%. Uptake rates reported for RCTs [29, 31, 32] ranged from 83.2 to 94.5%. The mean uptake across all three studies was 86.8%.

#### Adherence

Five studies [28, 29, 31–33] assessed adherence levels to ERSs among patients referred for mental health reasons. Adherence was measured as a binary outcome in all studies and defined as whether participants completed the ERS once they had attended. Participants who attended no ERS sessions were not included in adherence calculations. Adherence levels ranged from 36.7% [28] to 75.4% [29]. Three of the studies [28, 31, 33] used a leisure centre-based ERS, with adherence levels ranging from 36.7% [28] to 41.1% [31]. Mean adherence in these three studies was 39.3%. The other two studies [29, 32] used ERSs involving regular face-to-face consultations and telephone calls, and had adherence levels of 75.4% [29] and 59.6% [32]. The mean adherence across both studies was 71.7%. The two studies [28, 32] with the shortest ERSs (8-12 weeks) had a mean adherence of 45.8%. The study [29] with the longest ERS (32 weeks) had an adherence of 75.4%.

#### Adherence in routine practice

Two studies took place in routine practice [28, 33]. Adherence levels were 36.7% [28] and 37.6% [33]. Mean adherence across both studies was 37.2%. Both studies were leisure centre-based.

#### Adherence in trials

Three studies were RCTs [29, 31, 32]. Adherence levels ranged from 41.1 to 75.4% and the mean adherence across all studies was 55.3%. Two RCTs [29, 32] used ERSs involving regular face-to-face consultations and telephone calls. Adherence levels were 75.4 [29] and 59.6% [32]. Mean adherence across both studies was 71.7%. The other RCT [31] was leisure centre-based and had an adherence of 41.1%.

### Long-term physical activity levels

Two RCTs [29, 30] assessed the effects of ERSs on long-term physical activity levels among patients referred for mental health reasons (Table 6). Chalder et al. [29] asked participants to record physical activity levels in the week before assessment. These were converted into MET minutes [36] of physical activity per week (MET = metabolic equivalent of the task as a ratio to the basal rate). Murphy et al. [30] assessed exercise levels using the 7-day Physical Activity Recall Scale (7D-PAR) [37].

When combining results from 4, 8 and 12 months, Chalder et al. [29] recorded a significant between group difference in the number of patients doing ≥1000 MET minutes of physical activity per week in favour of the intervention group. After adjusting for all covariates, Murphy et al. [30] recorded a non-significant between group difference in 7D-PAR scores at 12 months in favour of the intervention group.

### Secondary outcomes

A secondary aim of this review was to assess differences in ERS uptake/adherence in mental health referrals compared to non-mental health referrals (Table 5).

### Uptake and adherence in mental health referrals vs. non-mental health referrals

Six studies [27, 28, 30, 33–35] assessed this outcome (Table 5). One of these was a RCT [31] and five were in a routine practice setting [27, 28, 33–35]. All comparator groups received the same ERS intervention as their respective mental health referral group.

### Uptake in mental health referrals vs. non-mental health referrals

Five studies [27, 28, 31, 34, 35] assessed uptake. Mental health referral uptake ranged from 42.3 to 83.2%, and comparator group uptake ranged from 27.1 to 85.8%. Four of these studies [27, 28, 31, 35] assessed leisure centre-based ERS. Uptake in leisure centre-based ERSs ranged from 54.5 to 83.2% for mental health referrals and 67.7–85.8% in comparator groups. Mean uptake across these four studies was 56.4% for all mental health referral participants and 71.2% for comparator groups. The other study [34] involved yoga classes and had an uptake of 42.3% for mental health referrals and 27.1% for the comparator group.

Four studies [27, 28, 34, 35] took place in routine practice. Uptake ranged from 42.3 to 82.6% in mental health referrals and 27.1–78.9% in comparator groups. Mean uptake across these studies was 55.3% for mental health referrals and 70.2% for comparator groups. Three of the studies in routine practice [27, 28, 35] were leisure centre-based. Uptake of mental health referrals in the leisure centre-based studies ranged from 54.4 to 82.6%, with a mean uptake of 55.5% across all three studies. Uptake of non-mental health referrals in the leisure centre-based studies ranged from 67.7 to 78.9%, with a mean uptake of 70.7% across all three studies. The other study based in routine practice [34] involved yoga classes and had an uptake of 42.3% for all mental health referrals and 27.1% for non-mental health referrals.

One study was a RCT [31]. Mental health referral uptake was 83.2% and comparator group uptake was 85.8%. This ERS was leisure centre-based.

Two studies [31, 35] performed between group statistical analyses. In a trial setting, Moore et al. [31] discovered a non-significant difference in favour of greater uptake in the comparator group. In a routine practice setting, Morgan et al. [35] discovered a significant difference in favour of greater uptake in the comparator group.

### Adherence in mental health referrals vs. non-mental health referrals

Three studies [28, 31, 33] assessed adherence to leisure centre-based ERS. Adherence ranged from 36.7 to 41.1% in mental health referrals and from 47.0 to 55.5% in comparator groups. Mean adherence across these studies was 39.3% for mental health referrals and 49.9% for comparator groups.

Two studies [28, 33] took place in routine practice. Mental health referral adherence levels were 36.7% [28] and 37.6% [33], with a mean adherence of 37.3% across both studies. Comparator group adherence levels were 48.8% in Crone et al. [28] and 47% in Tobi et al. [33], with a mean adherence of 48.4% across both studies.

One study [31] was a RCT. Mental health referral adherence was 41.1% and comparator group adherence was 55.5%.

Two of the studies [31, 33] performed between group statistical analyses. In a trial setting, Moore et al. [31] discovered a significant difference in favour of greater adherence in the comparator group. In a routine practice setting, Tobi et al. [33] also discovered a significant difference in favour of greater adherence in the comparator group.

## Discussion

The aims of this review were to evaluate: (1) clinical effectiveness of ERSs for mental health symptoms; (2) uptake and adherence of participants referred for mental health reasons in ERSs; (3) effects of ERSs on long-term physical activity levels in mental health participants. Uptake and adherence levels were also compared between mental health referrals and non-mental health referrals as a secondary outcome. This was to address the current evidence gap on this topic [16].

The short-term symptom improvement in ERS groups involving regular face-to-face consultations and telephone calls was not significant. Long-term improvement in symptoms for those taking part in leisure centre-based ERSs was statistically significant, however, it is important to emphasise this is based on the findings of a single study [30]. No leisure centre-based ERS studies assessed short-term clinical effectiveness. When combining studies in trial and routine practice settings, regular face-to-face consultations and telephone calls had the highest mean uptake and adherence levels [29, 32]. Only two studies [29, 30], both RCTs, measured the impact of ERSs on long-term physical activity levels in participants referred for mental health reasons. Regular face-to-face consultations and telephone calls [29] seemed to be more effective at increasing physical activity levels after 12 months than the leisure centre-based ERS [30].

Uptake and adherence to ERSs in mental health referrals was also compared to figures for uptake and adherence among those referred for other conditions. Although this comparison was not the primary aim of this review, it provided context and a point of reference for the uptake/adherence outcomes. Studies assessing both groups in leisure centre-based ERSs all recorded higher uptake [27, 28, 31, 35] and adherence [28, 31, 33] in non-mental health referrals. The yoga-based ERS [34] was the only study with higher uptake levels in mental health referrals. Nonetheless, uptake levels for mental health participants in this study were still lower than in any mental health referral group in leisure centre-based ERS.

There are several potential reasons as to why ERSs involving regular face-to-face consultations and telephone calls were found to be ineffective in improving symptoms compared to usual care in a control group [29, 32]. It is possible that the ERSs did not increase physical activity levels sufficiently to affect the symptoms [29]. Participants involved were also aware of their underlying condition and had voluntarily sought treatment. Additionally, trial participants are likely to already have greater motivation to change their lifestyle if they have agreed to take part in the study in the first place, whether they are allocated to the intervention or the control arm. Therefore, even though control groups received usual care, they may still have taken part in exercise or concurrently received other forms of effective treatment.

Both studies [29, 32] which involved regular face-to-face consultations and telephone calls were trials. It is difficult to say unequivocally whether the improved uptake and adherence levels in these studies were due to the type of ERS undertaken or the study setting. Nevertheless, both studies [29, 32] did achieve greater uptake and adherence levels than the one leisure centre-based trial [31].

Avery et al. [34] was the only study with a higher level of uptake in mental health referrals compared to non-mental health referrals, but it was nevertheless lower than in any of the mental health referral groups in leisure centre-based ERSs [27, 28, 31, 35]. However, since only army veterans were allowed to sign up, the patient population is not directly comparable to the other studies. Therefore, although mental health referral uptake levels were lower than in other studies, the fact that mental health participants were more likely to attend the yoga classes than participants referred for physical health reasons merits further exploration. One possible explanation is that the more mindful and meditative nature of yoga makes it more appealing than standard gym sessions for people with mental health conditions.

Of those studies that collected data on uptake and adherence within both mental health referrals and non-mental health referrals [27, 28, 31, 33–35], only two [28, 33] provided information on the mean ages for both groups. In both studies, mental health referrals had a lower level of uptake [28] and adherence [28, 33], but both also recorded a lower mean age in this group than their physical health counterparts. Other studies not included in this review have found that increasing age is positively correlated with greater ERS uptake levels [38–40]. This suggests that age could be acting as a confounding factor in mental health referrals and may be partly responsible for the lower levels of uptake and adherence in this group. It is also important to stress that most ERSs were designed to help those with chronic physical health conditions, which usually have an older age of onset compared with mental health conditions [28, 41, 42]. Tobi et al. [33] showed that people who were referred to ERSs for reasons of mental ill health were likely to be younger than those referred for physical health reasons. The researchers found that older participants referred to the schemes because of their mental health diagnoses were less likely to drop out than younger participants, and older males were the more likely to complete the programmes [33]. The relationship between the success of ERSs for those with mental health referrals and patient age merits further research.

### Results in the context of previous research and implications for policy and practice

This is the first review to look at ERSs and mental health in participants referred specifically for mental health reasons. Previous reviews have indicated that ERSs are beneficial to both mental health [20, 21] and psychological wellbeing [17], but these have been part of wider reviews that have included other conditions [17, 20, 21]. Some of the included studies assessed mental health symptoms in those participants referred for non-mental health reasons [43, 44]. Although not based in a primary care setting, one study found that ERSs ameliorated symptoms of male prisoners referred for mental health treatment [45].

The low uptake and adherence levels in mental health referrals suggests that the approach to ERSs within this population needs to change, with the standard leisure centre ERSs seemingly not having the same acceptability as it does for non-mental health referrals. Within the trial-based studies assessing uptake and adherence [29, 31, 32], ERSs involving regular face-to-face consultations and telephone calls [29, 32] had greater levels of uptake and adherence compared to leisure centre-based ERSs [31]. This could signify one-to-one meetings with health professionals (with no instant exercise obligations), are generally more appealing and less daunting to individuals presenting with mental health conditions. Both studies [29, 32] also adopted motivational interview techniques during meetings, suggesting that it may be beneficial to incorporate this into future ERS. Previous research supports this conclusion. Busch et al. [46] discovered that the majority of depressed individuals would be interested in exercise programmes, but see their depressive symptoms as a barrier, whilst Rouse et al. [47] found that autonomy support significantly improved intrinsic motivation. Screening patients for motivation levels before referral could also be beneficial in assessing suitability. ERSs should also be engaging and individualised to patients, as it has been shown that higher levels of attendance are associated with participant satisfaction with such interventions rather than the degree of severity of depression [48].

Flexibility in the delivery of the ERSs could improve participants’ level of satisfaction and engagement. This is particularly important in the context of mental health, in which the cyclical nature of conditions, such as depression, is likely to result in setbacks [49]. Research has investigated factors that affect the decisions of those with serious mental illness to initiate physical activity. It highlighted the particular importance of participants’ autonomy to decide their levels of participation in the activity [19]. Participants also considered it beneficial to know beforehand what they should expect the activity to entail and that it could be adapted to their needs [19]. Such knowledge, contributed to a supportive atmosphere, which was required to make exercise a success in this population. This all points towards the requirements that ERSs be tailored, individualised and personalised for people referred for mental health reasons [28], and that activities should be designed specifically for this group. An individualised and more holistic approach would enable consideration of aspects such as social circumstances, motivation, the availability of support, and cost [19, 49]. Schemes should prioritise promoting enjoyment and the promotion of autonomy through joint decision making in the early stages of the physical activity [19, 49]. These findings strengthen the argument made in this review that more mindful exercises such as yoga, which was used by Avery et al. [34], may be particularly beneficial for this patient group. Previous research indicating that yoga is beneficial for depression [50] and anxiety symptoms [51] further supports this assertion and strengthens the case for mindful exercise classes to be considered as part of ERS.

It has been shown that social support is required to help an individual with mental ill health to start physical activity in the community [19, 49, 52], and that this support should be provided by someone who is trusted and/or well-known to the individual [19, 53], such as health professionals [54], family members or friends [55]. The interpersonal relationship between the participant and their support team has been shown to play a big role in giving individuals with serious mental illness the confidence to start a new physical activity [19]. Previous research has also shown that individuals with depression who have several supportive social relationships show improved symptoms in response to the exercise treatment [56]. A non-judgemental, supportive atmosphere among peers and staff is particularly important for individuals with mental health conditions [19]. Formation of groups that are specifically tailored to the improvement of mental health could help to create the feeling of a shared identity among participants and responsibility towards others, which is known to be important for the engagement of people who attend group based physical activities [49, 52, 53, 57, 58].

The long-term physical activity results reported in Chalder et al. [29] suggest that having extra face-to-face consultations and telephone calls is more effective at encouraging individuals to maintain long-term physical activity levels, compared to leisure centre-based ERS programmes [30] with less frequent contact. Additional meetings may contribute to the support network of the participants. It is important to note, however, that this finding is only based on three studies and there were no routine practice studies assessing regular face-to-face consultations and telephone calls. Some of the leisure centre-based ERS studies already incorporate two face-to-face consultations [27, 30, 31, 35], but increasing this number further may improve adherence for mental health referrals.

Another important aspect, outside the scope of this review, is cost-effectiveness. Previous research suggests that ERSs are cost-effective for fully adherent participants [21]. Individual RCTs have also indicated that leisure centre-based ERSs for mental health referrals [59] and walking programmes for depression [60] can be cost-effective. This shows ERSs are a viable approach for managing patients who present with mental health conditions in primary care, but it is important to find the ERS programmes with the best symptom control and uptake to achieve optimum value for money. Participants may be less inclined to take up and maintain physical activity if there is a financial cost [19, 49]. Low socio-economic status of participants has been found to have a negative effect on their uptake, adherence to and completion of the schemes, irrespective of the primary reason for referral [35, 61]. Regardless of whether monetary support is provided to help an individual to initiate the activity, that activity must be affordable in the long term for people to sustain their participation. Therefore, building an exercise support network for referred participants outside of paid classes could play a vital role in maintaining increased physical activity levels. The inclusion of motivational techniques may also help with initiation and maintenance of the schemes.

#### Strengths and limitations of evidence and review

A key strength of this review is the inclusion of both RCTs and studies undertaken in routine practice. The high internal validity of RCTs made the clinical effectiveness findings more reliable. However, RCTs may not be the most appropriate way to measure what uptake and adherence would be like in the real world. Data from routine practice have greater external validity, making this a better representation of the mental health population in primary care. It is therefore essential that a circumspect approach is taken when interpreting combined uptake and adherence results from RCTs and studies from routine practice.

The large amount of heterogeneity between studies made it difficult to evaluate ERS. There were different types, lengths and settings for ERS, and different outcome measures. Ideally, ERSs would be assessed according to these variables. Lack of research into clinical effectiveness and long-term physical activity levels in particular means this cannot be achieved.

The wide range of outcome measures made any direct comparison of these studies difficult. For clinical effectiveness, all three studies [29, 30, 32] used different measuring scales. Additionally, it is difficult to accurately measure physical activity levels. Both Murphy et al. [30] and Chalder et al. [29] used methods that only recorded physical activity in the week leading up to assessment, meaning participants may have increased exercise levels solely during this period. Not disclosing assessment timings would be one way to address this. The simple uptake definition of whether a participant attended a session after referral, made results for this outcome more reliable when collating and comparing data. However, different adherence definitions made this outcome less comparable. Chalder et al. [29] defined adherence as receiving ≥5 ERS sessions, which was under 50% of the 13 available. This is significantly less than the ≥80% attendance required in other studies to be classified as an adherer [28, 33], potentially explaining the higher levels of adherence recorded by Chalder et al. [29].

There were limitations in a number of the studies included. Blinding was an issue in RCTs [29–32] due to the nature of ERSs making this impossible. There was also a high risk of attrition bias, with numerous participants dropping out. Furthermore, participants in some studies did not provide reasons for withdrawal [29, 30, 32].

#### Areas for future research

There is a sizeable gap in the literature regarding trials assessing the effect of ERSs on mental health symptoms in those referred for mental health reasons. No published research was found to have investigated the short-term effectiveness of leisure centre-based ERSs on symptoms in participants referred for mental health reasons. This is important, considering that most ERSs are currently leisure centre based. More studies assessing ERSs involving individualised programmes and mindful exercises are also needed, as are RCTs comparing different types of ERSs for mental health referrals. Research is also required on the effects of ERSs on long-term physical activity in mental health referrals; longer follow ups in future trials could help achieve this.

## Conclusions

There is evidence, albeit limited, that leisure centre-based ERSs can improve long-term mental health symptoms in those referred for mental health reasons. Evidence also suggests that ERSs involving regular face-to-face consultations and telephone calls are more effective than leisure centre-based ERSs in terms of increasing uptake, adherence, and long-term physical activity levels; however, this type of programme has not been assessed in routine practice. Future research is required to explore what types of ERS are most clinically effective, including the consideration of mindful exercise options such as yoga. Services should also consider including more mindful exercise options to improve the quality of their provision.

Existing ERSs could be improved through application of more individual tailoring, motivational techniques, and the provision of more face-to-face consultations, and social support.

## Supplementary Information

**Additional file 1.** Original protocol. 

**Additional file 2.** Literature search strategy used in the databases of Medline, PsycInfo, CINAHL, Scopus, and the Cochrane Library.

**Additional file 3.** Reasons for exclusion of full-text articles during study selection stage.

**Additional file 4:** Quality assessment of included studies. **Figure S1.** Risk of bias graph for RCTs. **Figure S2.** Risk of bias summary for RCTs.

## Data Availability

All the data generated in this study is provided in the published article (Fig. 1; Tables 1, 2, 3, 4, 5 and 6) or the additional supporting files (Figure S1, Figure S2, Table S1, Table S2).

## Abbreviations

BDI-II: Beck Depression Inventory version II
CI: Confidence interval
DASS-21: Depression, Anxiety and Stress Scale – 21 items
ERS: Exercise referral scheme
HADS: Hospital Anxiety and Depression Scale
MET: Metabolic equivalent of task
NICE: National Institute for Health and Care Excellence
OR: Odds ratio
RCT: Randomised controlled trial
7D-PAR: 7-Day Physical Activity Recall Scale
