# Supplementary material for: Evaluation of the uptake, retention and effectiveness of exercise referral schemes for the management of mental health conditions in primary care: a systematic review
Source: BMC Public Health. 2022 Feb 7;22:249. doi: 10.1186/s12889-022-12638-7 (PMC8822691; doi:10.1186/s12889-022-12638-7)
Supplement: Supplementary file 1 — Additional file 1. Original protocol. [file 12889_2022_12638_MOESM1_ESM.docx]

**An Evaluation of the Uptake, Retention and Effectiveness of Exercise Referral Schemes for the Management of Mental Health Conditions in a Primary Care Setting: A Systematic Review Protocol.**

*S. Tomlinson-Perez*

**Background**

Depression and anxiety are the most common psychiatric disorders seen in the general population, (1) with depression having a prevalence of 4.5% in the UK (2) and approximately 25% of adults experiencing anxiety at some point in their life. (3) Depressive disorders are one of the leading causes of disability globally and are the leading cause of disability and premature deaths in adults aged 18-44. Depression is characterised by constant low mood and/or loss of enjoyment in the majority of activities, along with a range of related emotional, cognitive, physical and behavioural symptoms. (2) The classic presentation of generalized anxiety disorder is disproportionate, pervasive, uncontrollable and widespread worry, with potential somatic, cognitive and behavioural symptoms that can vary in severity. (3)

It is widely accepted that exercise and physical activity is beneficial for a person’s mental wellbeing. It has been shown to improve self-esteem and mood, whilst also being effective for mental health disorders such as clinical depression and anxiety. (4) Even though it is well known that exercise has health benefits, there are large numbers of people who lead sedentary lives. In England, 34% of men and 42% of women do not achieve the recommended amount of weekly aerobic exercise (150 minutes of moderate activity or 75 minutes of vigorous activity). (5) In addition, 27% of adults exercise for less than 30 minutes a week and are classified as inactive. (6) It is particularly difficult for people with severe mental illness to regularly participate in exercise, with low mood, stress and a lack of support being the most prevalent barriers to participation. (7)

In the UK, one method to increase physical activity levels for sedentary individuals is via exercise referral schemes. These consist of an assessment by primary care or allied health professionals to determine if someone is sedentary, followed by a referral to a physical activity specialist/service. The patient is then advised on the type of physical activity that suits their specific needs and given the opportunity to take part in an exercise programme. (8) These programmes are eligible to be funded by commissioners for the rehabilitation and management of certain health conditions such as myocardial infarctions, stroke, chronic heart failure, chronic obstructive pulmonary disease, low back pain and depression. (8)

It is important to know how effective exercise referral schemes can be for mental health conditions such as depression and anxiety and whether they can be a viable method of management. This includes looking at the uptake and dropout rate of patients with depression or anxiety, whether there are any improvements to their clinical symptoms throughout the programme and for a period afterwards, and whether individuals continue to stay active of their own accord after the conclusion of the programme.

A preliminary literature search has highlighted that there is a current gap in the literature surrounding exercise referral schemes and mental health conditions in primary care such as depression and anxiety. Previous reviews that have looked at the effect of these schemes on mental health as part of their wider effect on health (9, 10), however, there are no recent reviews that explore the clinical effectiveness, long term outcomes and uptake/dropout rates of these schemes specifically for mental health referrals.

**Aims and Objectives**

The overall aim of this systematic review will be to assess how well exercise referral schemes work as a management method for individuals with mental health conditions who are referred from primary care. The judgement of how effective these schemes are will be based on the following research questions:

1. What are the effects of exercise referral schemes on the clinical symptoms in individuals referred for mental health conditions from primary care?
2. What are the uptake and dropout rates for exercise referral schemes for individuals with mental health conditions referred from primary care?
3. Do exercise referral schemes promote an increase in long term physical activity levels for individuals with mental health conditions referred from primary care?

The primary objectives of this systematic review are:

1. To explore the existing evidence base regarding exercise referral schemes and individuals with depression or anxiety.
2. To identify potential factors that may impact upon the effectiveness of exercise referral schemes for depression and anxiety. Examples could include sex, ethnicity, age and social class.
3. To analyse and discuss the results of identified studies to come to a conclusion for the research topic.

A secondary objective is to assess uptake and adherence in mental health referrals compared to non-mental health referrals in included studies.

**Eligibility Criteria**

***Study design*** *–* All study design types will be eligible for the systematic review.

***Population*** – Study participants should be diagnosed with a mental health condition, with primary care being the main source of referral.

***Intervention*** – Studies should evaluate exercise referral schemes as defined by the National Institute for Health and Care Excellence (NICE). (8) Alternative names such as physical activity referral schemes will also be accepted.

***Comparator*** – Any control (e.g. usual care); no intervention; baseline mental health of individuals before intervention; other forms of exercise interventions; or other conditions (e.g. physical health) if assessing for participation rates.

***Outcomes*** – Studies should assess for changes in clinical symptoms of mental health conditions as a result of exercise referral schemes; or assess participation rates of individuals with mental health conditions in exercise referral schemes (including uptake and dropout rates); or assess impact of exercise referral schemes on long term physical activity levels.

Studies will be restricted to English Language and there will be no date limitations.

**Information sources**

To find studies for the review, electronic searches of 5 online databases will be performed. The databases searched will be MEDLINE, Scopus, the Cochrane Library, PsycINFO and CINAHL. Reference lists of studies selected for the review will then be scanned, in addition to reference lists from existing relevant systematic reviews. Citation searches of selected studies will then be conducted using Google Scholar.

**Search strategy and study selection**

A draft search strategy for Medline is presented below. Similar search terms and limits will be used for the other databases. Titles and abstracts will be scanned to exclude irrelevant studies and a deduplication process will also be carried out. Remaining studies will be read in full and selected for inclusion if they meet the eligibility criteria. Selected studies will also be assessed for potential inclusion in a meta-analysis. Two reviewers will be responsible for study selection, with a third reviewer consulted over any disagreements.

--------------------------------------------------------------------------------

1 exp Exercise/ or exp Exercise Therapy/ (226126)

2 exercise.tw. (257106)

3 physical activity.tw. (107045)

4 1 or 2 or 3 (436571)

5 exp "Referral and Consultation"/ (74637)

6 referral.tw. (97643)

7 5 or 6 (152931)

8 4 and 7 (2838)

9 exp Mental Health/ (38082)

10 mental health.tw. (141635)

11 exp Depression/ (118492)

12 depression.tw. (329781)

13 anxiety.tw. (188096)

14 exp Mood Disorders/ (121675)

15 exp Anxiety/ (84707)

16 exp Anxiety Disorders/ (79129)

17 9 or 10 or 11 or 12 or 13 or 14 or 15 or 16 (678055)

18 8 and 17 (284)

19 limit 18 to english language (277)

**Data collection**

Information on study characteristics and outcomes will be collected from selected studies using a piloted data extraction form. The following details will be recorded from each study:

- *Study details:* type of study, author, year of publication, funding sources, setting, recruitment and allocation process, eligibility criteria, mental health severity.
- *Participant detail*s: age, gender, ethnicity, social class and sample size.
- *Intervention/comparator detail*s: type, length, frequency, duration and setting of the exercise referral scheme sessions and comparator.
- *Outcome details:* primary and secondary outcomes, outcome measures, type of analysis, timing of outcome assessment.
- *Results:* uptake/dropout rates, short- and long-term clinical effects on mental health, continuation of exercise post-intervention.

If data is missing from a publication, the study authors will be contacted directly. This process will be undertaken by two reviewers, with a third reviewer consulted over any disagreements.

**Quality assessment**

Studies will be individually assessed for risk of bias. The method used to analyse risk of bias will be different depending on the study type. The Cochrane Collaboration’s risk of bias tool (11) will be used to analyse risk of bias in all included Randomised controlled trials (RCTs). Other types of study will be assessed using the preferred checklist according to NICE guidelines. (12) A risk of bias graph and summary table will be displayed consisting of all included studies.

**Data synthesis**

Studies will be pooled depending on the outcome being measured (e.g. mental health symptoms, uptake/dropout rates, long term physical activity levels) and the outcome measuring tool used. If possible, a meta-analysis will be performed to explore the effect of exercise referral schemes on mental health symptoms. For categorical outcome data, odds ratio will be used as the meta-analysis effect measure. For continuous outcome data, mean difference or standardised mean difference will be used as the effect measure depending on whether the same outcome measuring scales were used in studies. The meta-analysis will be performed with Review Manager 5.3 software. (13) Inter-study statistical heterogeneity will be calculated using Higgins I^2^ values. If statistical pooling is not possible, results will be displayed as a narrative synthesis.

**References**

1. Carek PJ, Laibstain SE, Carek SM. Exercise for the treatment of depression and anxiety. The International Journal of Psychiatry in Medicine. 2011;41(1):15-28.

2. NICE. Depression 2020 [Available from: https://cks.nice.org.uk/depression#!topicSummary.

3. NICE. Generalized anxiety disorder 2017 [Available from: https://cks.nice.org.uk/generalized-anxiety-disorder#!topicSummary.

4. Fox KR. The influence of physical activity on mental well-being. Public health nutrition. 1999;2(3a):411-8.

5. Scholes S, Neave A. Health Survey for England 2016: Physical activity in adults. Health and Social Care Information Centre Available online: <https://files> digital nhs uk/publication/m/3/hse16-adult-phy-act pdf (accessed on 12 February 2018). 2017.

6. Population Health Team, NHS Digital. Health Survey for England 2018 Adult’s health-related behaviours. National Statistics. 2019.

7. Firth J, Rosenbaum S, Stubbs B, Gorczynski P, Yung AR, Vancampfort D. Motivating factors and barriers towards exercise in severe mental illness: a systematic review and meta-analysis. Psychological medicine. 2016;46(14):2869-81.

8. NICE. Physical activity: exercise referral schemes: National Institute for Health and Care Excellence,; 2014 [

9. Rowley N, Mann S, Steele J, Horton E, Jimenez A. The effects of exercise referral schemes in the United Kingdom in those with cardiovascular, mental health, and musculoskeletal disorders: a preliminary systematic review. BMC public health. 2018;18(1):949.

10. Pavey T, Taylor A, Fox K, Hillsdon M, Anokye N, Campbell J, et al. Effect of exercise referral schemes in primary care on physical activity and improving health outcomes: systematic review and meta-analysis. Bmj. 2011;343:d6462.

11. Higgins JP, Altman DG, Gøtzsche PC, Jüni P, Moher D, Oxman AD, et al. The Cochrane Collaboration’s tool for assessing risk of bias in randomised trials. Bmj. 2011;343:d5928.

12. National Institute for Health and Care Excellence. Developing NICE guidelines: the manual. Appendix H: Appraisal checklists, evidence tables, GRADE and economic profiles: NICE; 2018.

13. Collaboration C. Review Manager (RevMan)[Computer Program] Version 5.2. 3. Copenhagen: The Nordic Cochrane Centre; 2012. HEALTH PSYCHOLOGY REVIEW. 2014;17.
