# Supplementary material for: Evaluation of the uptake, retention and effectiveness of exercise referral schemes for the management of mental health conditions in primary care: a systematic review
Source: BMC Public Health. 2022 Feb 7;22:249. doi: 10.1186/s12889-022-12638-7 (PMC8822691; doi:10.1186/s12889-022-12638-7)
Supplement: Supplementary file 2 — Additional file 2. Literature search strategy used in the databases of Medline, PsycInfo, CINAHL, Scopus, and the Cochrane Library. [file 12889_2022_12638_MOESM2_ESM.docx]

**Table S1**. Medline, PsycInfo, CINAHL, Scopus and Cochrane Library search strategy

| **Search strategy for Medline: 277 records**  1 exp Exercise/ or exp Exercise Therapy/ (226332)  2 exercise.tw. (257396)  3 physical activity.tw. (107292)  4 1 or 2 or 3 (437129)  5 exp "Referral and Consultation"/ (74673)  6 referral.tw. (97812)  7 5 or 6 (153124)  8 4 and 7 (2843)  9 exp Mental Health/ (38149)  10 mental health.tw. (141936)  11 exp Depression/ (118590)  12 depression.tw. (330216)  13 anxiety.tw. (188443)  14 exp Mood Disorders/ (121722)  15 exp Anxiety/ (84787)  16 exp Anxiety Disorders/ (79157)  17 9 or 10 or 11 or 12 or 13 or 14 or 15 or 16 (678910)  18 8 and 17 (284)  19 limit 18 to english language (277) |
| --- |
| **Search strategy for PsycInfo: 84 records**  1 exp Physical Activity/ or exp Exercise/ (41915)  2 exercise.tw. (51736)  3 physical activity.tw. (34317)  4 1 or 2 or 3 (82508)  5 exp professional referral/ (3503)  6 referral.tw. (20329)  7 5 or 6 (21101)  8 4 and 7 (321)  9 exp Mental Health/ (66332)  10 mental health.tw. (189403)  11 exp affective disorders/ (143490)  12 exp major depression/ (131207)  13 depression.tw. (254082)  14 exp anxiety disorders/ (53980)  15 anxiety.tw. (198513)  16 exp Anxiety/ (71530)  17 9 or 10 or 11 or 12 or 13 or 14 or 15 or 16 (580518)  18 8 and 17 (84)  19 limit 18 to english language (84) |
| **Search strategy for CINAHL: 334 records**   \| **#** \| **Query** \| **Limiters/Expanders** \| **Results** \| \| --- \| --- \| --- \| --- \| \| S6 \| (mental health OR ( depression or depressive disorder or depressive symptoms or major depressive disorder ) OR ( anxiety disorders or anxiety or generalized anxiety disorder ) OR anxiety) AND (S3 AND S4) \| Expanders - Apply equivalent subjects Narrow by Language: - english Search modes - Boolean/Phrase \| 334 \| \| S5 \| (mental health OR ( depression or depressive disorder or depressive symptoms or major depressive disorder ) OR ( anxiety disorders or anxiety or generalized anxiety disorder ) OR anxiety) AND (S3 AND S4) \| Expanders - Apply equivalent subjects Search modes - Boolean/Phrase \| 338 \| \| S4 \| mental health OR ( depression or depressive disorder or depressive symptoms or major depressive disorder ) OR ( anxiety disorders or anxiety or generalized anxiety disorder ) OR anxiety \| Expanders - Apply equivalent subjects Search modes - Boolean/Phrase \| 373,391 \| \| S3 \| (exercise referral schemes OR physical activity referral OR referral) AND (S1 AND S2) \| Expanders - Apply equivalent subjects Search modes - Boolean/Phrase \| 2,376 \| \| S2 \| exercise referral schemes OR physical activity referral OR referral \| Expanders - Apply equivalent subjects Search modes - Boolean/Phrase \| 76,675 \| \| S1 \| exercise OR physical activity \| Expanders - Apply equivalent subjects Search modes - Boolean/Phrase \| 255,595 \| |
| **Search strategy for Scopus: 488 records**  ( ( exercise  AND referral  AND scheme )  OR  ( physical  AND activity  AND referral  AND scheme ) )  AND  ( ( mental  AND health )  OR  depression  OR  anxiety )  AND NOT  INDEX ( medline )  AND  ( LIMIT-TO ( LANGUAGE ,  "English" ) ) |
| **Search strategy for Cochrane Library: 439 records**   1. (Exercise referral):ti,ab,kw OR (physical activity referral):ti,ab,kw (Word variations have been searched) 1407 2. (mental health):ti,ab,kw OR (depression):ti,ab,kw OR (anxiety):ti,ab,kw (Word variations have been searched) 124053 3. #1 AND #2 439 |
