# Supplementary material for: Evaluation of the uptake, retention and effectiveness of exercise referral schemes for the management of mental health conditions in primary care: a systematic review
Source: BMC Public Health. 2022 Feb 7;22:249. doi: 10.1186/s12889-022-12638-7 (PMC8822691; doi:10.1186/s12889-022-12638-7)
Supplement: Supplementary file 3 — Additional file 3. Reasons for exclusion of full-text articles during study selection stage. [file 12889_2022_12638_MOESM3_ESM.docx]

Reasons for exclusion of full text articles

Reasons for exclusion:

- Different outcomes (n=20), *i.e. does not assess clinical effectiveness, uptake/adherence or long-term physical activity levels.*
- Different participant eligibility criteria (n=8)
- Did not meet ERS criteria (n=7)
- Mental health not primary referral reason (n=4)
- Qualitative (n=2)
- Not primary research (n=1)
- Study results reported elsewhere (n=1)

1. **Orchard JW. Prescribing and dosing exercise in primary care. Australian Journal Of General Practice. 2020;49(4):182-6.**

Reason for exclusion: different outcomes

Outcomes are about measuring exercise as a treatment method. There is no uptake/adherence analysis or analysis of mental health outcomes. There is some mention of depression but this is not explored directly.

2. **Nau T, Nolan G, Smith BJ. Enhancing Engagement With Socially Disadvantaged Older People in Organized Physical Activity Programs. International Quarterly of Community Health Education. 2019;39(4):257-67.**

Reason for exclusion: qualitative research

Outcomes are qualitative and structured through interviews. Only research with primarily quantitative data are included in this systematic review.

3. **McGeechan GJ, Phillips D, Wilson L, Whittaker VJ, O'Neill G, Newbury-Birch D. Service Evaluation of an Exercise on Referral Scheme for Adults with Existing Health Conditions in the United Kingdom. International Journal of Behavioral Medicine. 2018;25(3):304-11.**

Reason for exclusion: different outcomes

Outcomes are related to the amount of exercise conducted and the subject’s waist circumference and BMI. There are no mental health outcomes. There is data for uptake/adherence but not for mental health subjects specifically.

4. **Bartlem KM, Bowman J, Freund M, Wye PM, Barker D, McElwaine KM, et al.** **Effectiveness of an intervention in increasing the provision of preventive care by community mental health services: a non-randomized, multiple baseline implementation trial. Implementation Science. 2016;11:46.**

Reason for exclusion: different outcomes

Outcomes are related to client health behaviour risk status and the effect on their risk behaviours, not mental health symptoms or outcomes. Uptake/adherence is not clearly measured.

5. **Duda JL, Williams GC, Ntoumanis N, Daley A, Eves FF, Mutrie N, et al. Effects of a standard provision versus an autonomy supportive exercise referral programme on physical activity, quality of life and well-being indicators: a cluster randomised controlled trial. International Journal of Behavioral Nutrition and Physical Activity. 2014;11(1):10.**

Reason for exclusion: different outcomes

Indicators of mental health are offered as an outcome but results are not displayed for participants with mental health as their primary reason for referral.

6. **Rouse PC, Ntoumanis N, Duda JL, Jolly K, Williams GC. In the beginning: role of autonomy support on the motivation, mental health and intentions of participants entering an exercise referral scheme. Psychology & Health. 2011;26(6):729-49.**

Reason for exclusion: different outcomes

Outcomes are measuring the intentions of participants to engage in the exercise programme before the study rather than assessing the results after the programme.

7. **Raine P, Truman C, Southerst A. The development of a community gym for people with mental health problems: Influences on psychological accessibility. Journal of Mental Health. 2002;11(1):43-53.**

Reason for exclusion: different outcomes

Outcomes are qualitative in nature and are based on the service development, not the clinical outcomes of the patients or uptake/adherence.

8. **Fisher KJ, Li F. A community-based walking trial to improve neighborhood quality of life in older adults: a multilevel analysis. Annals of Behavioral Medicine. 2004;28(3):186-94.**

Reason for exclusion: different outcomes

Outcomes are not specific to a group suffering from mental health. Mental health is not the primary reason for referrals. Primary care is not the main source of referrals.

9. **Bilderbeck AC, Brazil IA, Farias M. Preliminary evidence that yoga practice progressively improves mood and decreases stress in a sample of UK prisoners. Evidence-Based Complementary and Alternative Medicine. 2015;2015.**

Reason for exclusion: different outcomes

Mental health symptoms are an outcome but not specifically for patients with mental health as the primary referral reason. The study setting is also in prisons, not primary care.

10. **Hardage J, Peel C, Morris D, Graham C, Brown CJ, Foushee HR, et al. Adherence to Exercise Scale for Older Patients (AESOP): a measure for predicting exercise adherence in older adults after discharge from home health physical therapy. Journal of Geriatric Physical Therapy. 2007;30(2):69-78.**

Reason for exclusion: different outcomes

Outcomes are looking at aspects that affect adherence but adherence is not measured directly.

11. **Messina E, Yoshitaka I. Analysis of Participation Levels in Activity Programming at a Correctional Mental Health Facility. Therapeutic Recreation Journal. 2013;47(3):197-211.**

Reason for exclusion: different outcomes

Outcomes are related to factors measuring participation, not uptake/adherence itself. Not based in primary care.

12. **Bailey D, Kerlin L. Can Health Trainers Make a Difference With Difficult-to-Engage Clients? A Multisite Case Study. Health Promotion Practice. 2015;16(5):756-64.**

Reason for exclusion: different outcomes

Data on the outcomes for mental health category is not clearly defined. Not based in primary care.

13. **Forsyth A, Deane FP, Williams P. Dietitians and exercise physiologists in primary care: lifestyle interventions for patients with depression and/or anxiety. Journal of Allied Health. 2009;38(2):e-63.**

Reason for exclusion: different outcomes

Study uses same data set as one of the included studies in the review. Outcomes are different and less preferable in this paper.

14. **Stewart L, Dolan E, Carver P, Swinton PA. Per-protocol investigation of a best practice exercise referral scheme. Public Health (Elsevier). 2017;150:26-33.**

Reason for exclusion: different outcomes

Mental health patients are included in the review but there is no specific data for this group reagrding adherence or symptoms.

15. **Forsyth A, Williams P, Deane FP. Physical activity, but not fitness level, is associated with depression in Australian adults. Journal of Sports Medicine & Physical Fitness. 2015;55(7/8):845-54.**

Reason for exclusion: different outcomes

Outcomes measure physical activity levels, however the number of people taking up or adhering to long-term physical activity levels is not studied.

16. **Zanetidou S, Belvederi Murri M, Menchetti M, Toni G, Asioli F, Bagnoli L, et al. Physical Exercise for Late-Life Depression: Customizing an Intervention for Primary Care. Journal of the American Geriatrics Society. 2017;65(2):348-55.**

Reason for exclusion: does not meet the ERS criteria

Does not fit ERS criteria outlined in this systematic review.

17. **Gusi N, Reyes MC, Gonzalez-Guerrero JL, Herrera E, Garcia JM. Cost-utility of a walking programme for moderately depressed, obese, or overweight elderly women in primary care: a randomised controlled trial. BMC public health. 2008;8(1):231.**

Reason for exclusion: different outcomes

Outcomes are looking at healthcare costs and cost-effectiveness of the ERS, not clinical symptoms or uptake/adherence.

18. **Sowden SL, Breeze E, Barber J, Raine R. Do general practices provide equitable access to physical activity interventions? British Journal of General Practice. 2008;58(555):e1-e8.**

Reason for exclusion: different outcomes

Participants with mental health conditions are included but there are no data outcomes specifically for the group referred for mental health reasons.

19. **James DV, Johnston LH, Crone D, Sidford AH, Gidlow C, Morris C, et al. Factors associated with physical activity referral uptake and participation. Journal of Sports Sciences. 2008;26(2):217-24.**

Reason for exclusion: different outcomes

Uptake for mental health participants is measured but data is only compared to participants referred for cardiovascular disease as an odds ratio. No separate data is presented for mental health participants.

20. **Tobi P, Estacio EV, Yu G, Renton A, Foster N. Who stays, who drops out? Biosocial predictors of longer-term adherence in participants attending an exercise referral scheme in the UK. BMC Public Health. 2012;12(1):347.**

Reason for exclusion: different outcomes

A study using the same dataset is included in the review. This paper displays the adherence of mental health participants in less detail than the study included in the review.

21. **O’Toole S, Maguire J, Murphy P. The efficacy of exercise referral as an intervention for Irish male prisoners presenting with mental health symptoms. International Journal of Prisoner Health. 2018.**

Reason for exclusion: does not meet participant eligibility criteria

Participants are referred from a prison, not primary care.

22. **Daley A, Winter H, Grimmett C, McGuinness M, McManus R, MacArthur C. Feasibility of an exercise intervention for women with postnatal depression: a pilot randomised controlled trial. British Journal of General Practice. 2008;58(548):178-83.**

Reason for exclusion: does not meet participant eligibility criteria

The main source of referral is from the psychiatric mother and baby unit (secondary care), not from primary care.

23. **Pardo A, Violán M, Cabezas C, García J, Miñarro C, Rubinat M, et al. Effectiveness of a supervised physical activity programme on physical activity adherence in patients with cardiovascular risk factors. Apunts Medicina de l'Esport. 2014;49(182):37-44.**

Reason for exclusion: does not meet participant eligibility criteria

Participants are referred for cardiovascular reasons not mental health reasons.

24. **Ohlsen RI, Peacock G, Smith S. Developing a service to monitor and improve physical health in people with serious mental illness. Journal of Psychiatric & Mental Health Nursing (Wiley-Blackwell). 2005;12(5):614-9.**

Reason for exclusion: does not meet participant eligibility criteria

Referrals are made from nurse-led services (secondary care) not primary care.

25. **Taylor AH, Fox KR. Effectiveness of a primary care exercise referral intervention for changing physical self-perceptions over 9 months. Health Psychology. 2005;24(1):11-21.**

Reason for exclusion: does not meet participant eligibility criteria

Participants are not referred with mental health as the primary referral reason. Subsequently, outcomes are not related to uptake/adherence or changes in mental health symptoms.

26. **Taylor AH, Doust J, Webborn N. Randomised controlled trial to examine the effects of a GP exercise referral programme in Hailsham, East Sussex, on modifiable coronary heart disease risk factors. Journal of Epidemiology & Community Health. 1998;52(9):595-601.**

Reason for exclusion: does not meet participant eligibility criteria

Mental health is not the primary referral reason for participants.

27. **James EL, Ewald BD, Johnson NA, Stacey FG, Brown WJ, Holliday EG, et al. Referral for expert physical activity counseling: a pragmatic RCT. American Journal of Preventive Medicine. 2017;53(4):490-9.**

Reason for exclusion: different outcomes

Some paricipants are referred for depression, but there is no data on uptake/adherence specifically for these participants.

28. **Prior F, Coffey M, Robins A, Cook P. Long-Term Health Outcomes Associated With an Exercise Referral Scheme: An Observational Longitudinal Follow-Up Study. Journal of Physical Activity & Health. 2019;16(4):288-93.**

Reason for exclusion: does not meet participant eligibility criteria

Mental health is not the primary referral reason for participants.

29. **Midtgaard J, Stage M, Moller T, Andersen C, Quist M, Rorth M, et al. Exercise may reduce depression but not anxiety in self-referred cancer patients undergoing chemotherapy. Post-hoc analysis of data from the 'Body & Cancer' trial. Acta Oncologica. 2011;50(5):660-9.**

Reason for exclusion: does not meet participant eligibility criteria

Referrals are not made from primary care.

30. **Helgadóttir B, Hallgren M, Kullberg CL, Forsell Y. Sticking with it? Factors associated with exercise adherence in people with mild to moderate depression. Psychology of Sport and Exercise. 2018;35:104-10.**

Reason for exclusion: does not meet the ERS criteria

The ERS intervention is not tailored to an individual’s requirements.

31. **Krogh J, Lorentzen AK, Subhi Y, Nordentoft M. Predictors of adherence to exercise interventions in patients with clinical depression–a pooled analysis from two clinical trials. Mental Health and Physical Activity. 2014;7(1):50-4.**

Reason for exclusion: not primary research

This study is formed by the pooling of two clinical trials. Only studies with primary research are included in this systematic review.

32. **van Straten A, Cuijpers P, Smits N. Effectiveness of a web-based self-help intervention for symptoms of depression, anxiety, and stress: randomized controlled trial. Journal of Medical Internet Research. 2008;10(1):e7-e.**

Reason for exclusion: does not meet the ERS criteria

ERS is done via self-help methods and is not an exercise referral scheme in the community.

33. **Sadeghi K, Ahmadi SM, Ahmadi SM, Rezaei M, Miri J, Abdi A, et al. A comparative study of the efficacy of cognitive group therapy and aerobic exercise in the treatment of depression among the students. Glob J Health Sci. 2016;8(10):54171.**

Reason for exclusion: does not meet the ERS criteria

Participants are instructed to exercise, but there is no distinct ERS service that the students are referred to.

34. **Lord J, Green F. Exercise on prescription: does it work? Health Education Journal. 1995;54(4):453-64.**

Reason for exclusion: does not meet the ERS criteria

There is no monitoring of participants during the ERS programme.

35. **Murri MB, Amore M, Menchetti M, Toni G, Neviani F, Cerri M, et al. Physical exercise for late-life major depression. The British Journal of Psychiatry. 2015;207(3):235-42.**

Reason for exclusion: did not meet the ERS criteria

Exercise intervention is not individualised to participants.

36. **Bombardier CH, Ehde DM, Gibbons LE, Wadhwani R, Sullivan MD, Rosenberg DE, et al. Telephone-based physical activity counseling for major depression in people with multiple sclerosis. Journal of Consulting & Clinical Psychology. 2013;81(1):89-99.**

Reason for exclusion: does not have mental health as a primary referral reason

Participants have both multiple sclerosis and major depressive disorder as the primary reason for referral.

37. **Isaacs A, Critchley J, Tai SS, Buckingham K, Westley D, Harridge S, et al. Exercise Evaluation Randomised Trial (EXERT): a randomised trial comparing GP referral for leisure centre-based exercise, community-based walking and advice only. HEALTH TECHNOLOGY ASSESSMENT-SOUTHAMPTON-. 2007;11(10).**

Reason for exclusion: does not have mental health as a primary referral reason

Participants are selected based on the fact that they have a cardiovascular risk factor not mental health symptoms.

38. **Johnson NA, Ewald B, Plotnikoff RC, Stacey FG, Brown WJ, Jones M, et al. Predictors of adherence to a physical activity counseling intervention delivered by exercise physiologists: secondary analysis of the NewCOACH trial data. Patient preference and adherence. 2018;12:2537.**

Reason for exclusion: does not have mental health as a primary referral reason

There are some participants who have concomitant depression with their chronic disease but mental health is not the primary reason for referral.

39. **Edmunds J, Ntoumanis N, Duda JL. Adherence and well-being in overweight and obese patients referred to an exercise on prescription scheme: A self-determination theory perspective. Psychology of Sport and Exercise. 2007;8(5):722-40.**

Reason for exclusion: does not have mental health as a primary reason for referral

Participants are selected for fullfilling the overweight and obese criteria, mental health is not the primary reason for referral.

40. **Hanson CL, Oliver EJ, Dodd-Reynolds CJ, Allin LJ. How do participant experiences and characteristics influence engagement in exercise referral? A qualitative longitudinal study of a scheme in Northumberland, UK. BMJ Open. 2019;9(2):e024370.**

Reason for exclusion: qualitative research

No quantitative data is provided for the outcomes of this systematic review.

41. **Hefferon K, Mallery R, Gay C, Elliott S. ‘Leave all the troubles of the outside world’: a qualitative study on the binary benefits of ‘Boxercise’for individuals with mental health difficulties. Qualitative research in sport, exercise and health. 2013;5(1):80-102.**

Reason for exclusion: qualitative research

No quantitative data is provided on uptake/adherence or changes in mental health symptoms.

42. **Maier J, Jette S. Promoting Nature-Based Activity for People With Mental Illness Through the US "Exercise Is Medicine" Initiative. American Journal of Public Health. 2016;106(5):796-9.**

Reason for exclusion: not primary research

This article is a review of other literature. Only studies with primary research are included in this systematic review.

43. **Edwards RT, Linck P, Hounsome N, Raisanen L, Williams N, Moore L, et al. Cost-effectiveness of a national exercise referral programme for primary care patients in Wales: results of a randomised controlled trial. BMC Public Health. 2013;13(1):1021.**

Reason for exclusion: study results reported elsewhere

Same data set is used in a study (Murphy et al) that has been included in this systematic review.
