# Supplementary material for: Evaluation of the uptake, retention and effectiveness of exercise referral schemes for the management of mental health conditions in primary care: a systematic review
Source: BMC Public Health. 2022 Feb 7;22:249. doi: 10.1186/s12889-022-12638-7 (PMC8822691; doi:10.1186/s12889-022-12638-7)
Supplement: Supplementary file 4 — Additional file 4: Quality assessment of included studies. Figure S1. Risk of bias graph for RCTs. Figure S2. Risk of bias summary for RCTs. [file 12889_2022_12638_MOESM4_ESM.docx]

*Figure S1. Risk of bias graph for RCTs.*


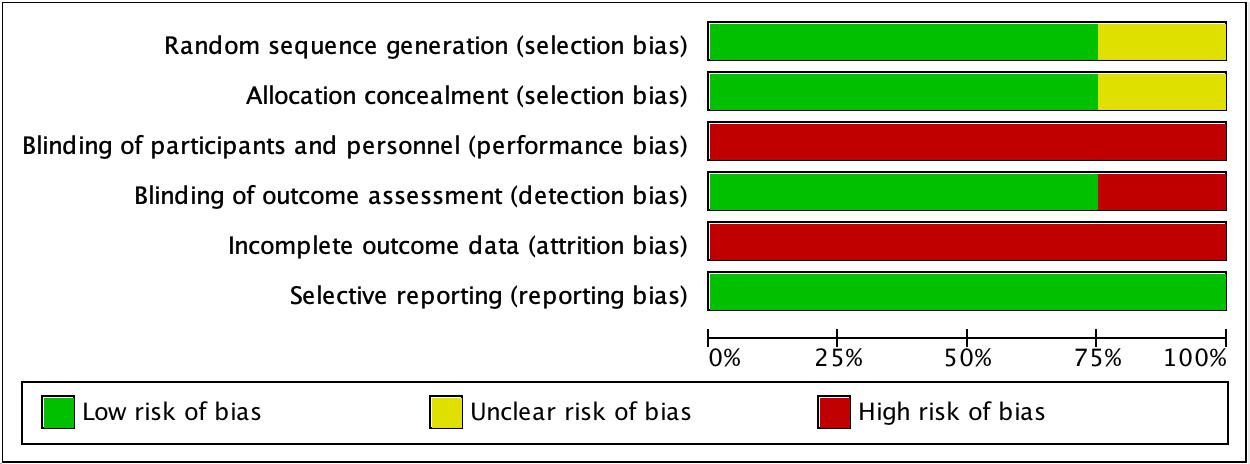


*Figure S2. Risk of bias summary for RCTs.*


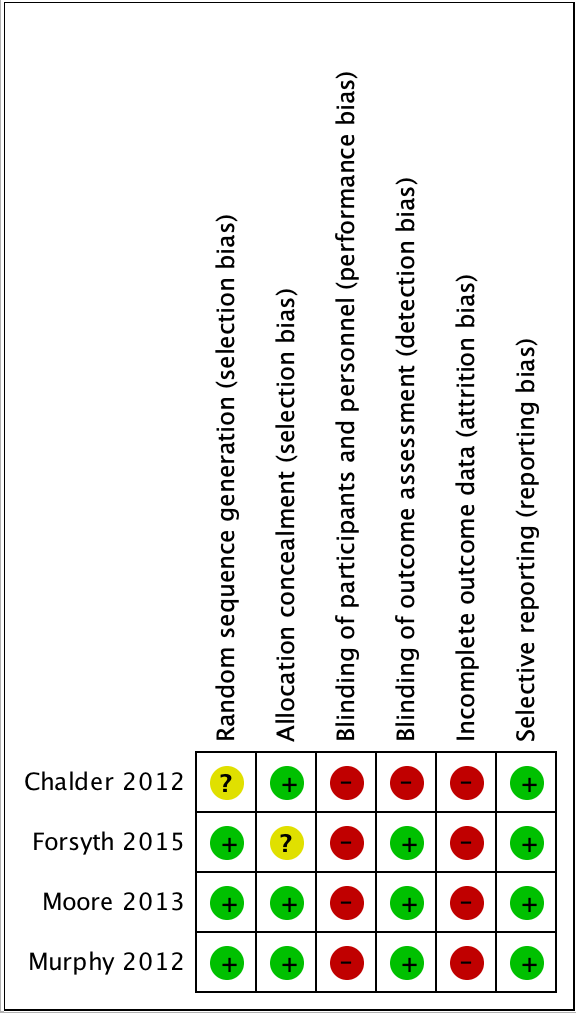


Table S2. Quality appraisal checklist for case series studies

| **Criteria** | **Harrison 2005** | **Crone 2008** | **Tobi**  **2017** | **Avery 2020** | **Morgan 2020** |
| --- | --- | --- | --- | --- | --- |
| *Clear study objective* | Yes | Yes | Yes | Yes | Yes |
| *Conducted prospectively* | No | No | No | No | No |
| *Cases collected from multiple centres* | Yes | Yes | Yes | Yes | Yes |
| *Patients recruited consecutively* | Unclear | Unclear | Unclear | Unclear | Unclear |
| *Description of patient characteristics* | Yes | Partial | Yes | Yes | Yes |
| *Clear eligibility criteria* | Partial | No | Partial | Yes | Yes |
| *Similar disease severity at study entry* | Unclear | Unclear | Unclear | Unclear | Unclear |
| *Clearly described intervention* | Yes | Partial | Partial | Yes | Yes |
| *Pre-established outcome measures* | Yes | Yes | Yes | Yes | Yes |
| *Appropriate methods for measuring outcome* | Yes | Yes | Yes | Yes | Yes |
| *Appropriate length of follow up* | Yes | Yes | Yes | Yes | Yes |
| *Loss to follow up reported* | Yes | Yes | Yes | Yes | Yes |
| *Results support study conclusions* | Yes | Yes | Yes | Yes | Yes |
| *Competing interests and support sources reported* | No | No | Partial | Partial | Yes |
